# Association of body roundness index with cardiovascular disease in patients with cardiometabolic syndrome: a cross-sectional study based on NHANES 2009-2018

**DOI:** 10.3389/fendo.2025.1524352

**Published:** 2025-02-03

**Authors:** Xiaohua He, Jingling Zhu, Wenfei Liang, Xiuling Yang, Weimin Ning, Zhan Zhao, Jingyi Chen, Qiuxing He

**Affiliations:** Department of Neurology, Dongguan Hospital of Guangzhou University of Chinese Medicine, Dongguan, China

**Keywords:** cardiometabolic syndrome, cardiovascular disease, body roundness index, NHANES, obesity

## Abstract

**Background:**

Cardiometabolic syndrome (CMS), marked by abdominal obesity and metabolic dysregulation, is associated with a heightened risk of cardiovascular disease (CVD). Compared to the traditional anthropometric predictors represented by body mass index (BMI) and waist circumference (WC), body roundness index (BRI) appears to provide a more accurate reflection of the abdominal fat distribution associated with metabolic diseases. Therefore, this study intends to investigate the association of BRI with the risk of CVD and its components including congestive heart failure (CHF), coronary heart disease (CHD), angina, heart attack, and stroke in patients with CMS. At the same time, we hypothesized that BRI would identify CVD better than BMI or WC.

**Methods:**

Data from the 2009-2018 National Health and Nutrition Examination Survey (NHANES). Logistic regression models were mainly utilized to evaluate the relationship between BRI and CVD in patients with CMS, including smooth curve analysis, threshold effects analysis, subgroup analysis and multiple imputation. In addition, receiver operating characteristic (ROC) curves were used to assess the ability of BRI to predict CVD.

**Results:**

The logistic regression model showed a positive association between the BRI and CVD. The highest quartile of BRI (Q4) showing the strongest association with CVD. The smoothed curve revealed a linear relationship between BRI and CVD, but a U-shaped association between the BRI and CHF. For CVD, stratified analyses did not show significant difference between strata. For CHF, BMI interacted with the association, with BRI being associated with decreased risk of CHF in a subgroup of normal weight subjects and increased risk of CHF in a subgroup of obese subjects. The multiple imputation further confirmed the robustness of these results. Additionally, the ROC curve indicated that BRI, BMI and WC had predictive power for CVD and CHF (AUC > 0.05). BRI has similar predictive power to WC but better than BMI.

**Conclusions:**

An elevated BRI is associated with a heightened risk of CVD in patients with CMS. BRI has similar ability to predict CVD and CHF as WC, but superior to BMI.

## Introduction

Cardiovascular disease (CVD) is recognized as a significant public health concern due to its high prevalence, morbidity, and mortality rates globally (1, 2). The global burden of CVD is expected to increase, primarily driven by an aging population (3). A study examining the global burden of disease from 1990 to 2019 noted a rise in CVD cases from 271 million to 523 million, along with a marked increase in mortality rates (4). Cardiometabolic factors make one of the major contributors to the risk of CVD. Hypertension, hyperlipidemia, diabetes and obesity are modifiable metabolic risk factors for CVD (5, 6). Hence, it is important to identify and screen people at risk for CVD at an early stage, especially those with metabolic risk factors, and to implement timely interventions.

Cardiometabolic syndrome (CMS) serves as a significant risk factor for CVD. It contributes to heightened cardiovascular and all-cause mortality rates (7). CMS is defined by abdominal obesity, hypertension, increased triglycerides, reduced levels of high-density lipoprotein cholesterol (HDL-C), and glucose intolerance (8, 9). Typically, the diagnosis of CMS demands the existence of at least three of these risk factors. As the population ages, the global metabolic risk is rising, leading to an increased prevalence of CMS between 1999 and 2018 (10), the prevalence of CMS among American adults rose from 28.23% to 37.09%, suggesting a deterioration in cardiometabolic health status (11). Alarmingly, merely 6.8% of adults preserve optimal cardiometabolic well-being (12). It is widely recognized that excessive obesity, particularly centripetal obesity, independently intensifies a multitude of metabolic risk factors for CVD, including the induction of dyslipidemia, increased blood pressure, hyperglycemia, insulin resistance (IR), and systemic inflammation (5, 13). Obesity may also lead to an increased risk of cardio-metabolic disease in children and adolescents (14).

Obesity is identified as a primary contributor to the burden of CMS (11, 15, 16). Moreover, the incidence of CVD is significantly higher in obese people, and the excessive accumulation of body fat leads to the development of CVD by inducing an inflammatory response and increasing oxidative stress (17). Obesity is increasingly being used to identify people at risk for cardiovascular risk factors, and notably abdominal obesity is a strong risk factor for CVD. Thomas proposed a novel body roundness index (BRI) that estimates the rate of visceral fat to total body fat by combining waist circumference (WC) and height data (18). Traditional research methods frequently depend on indicators like body mass index (BMI) and WC for the identification and management of obesity (19, 20). However, they all have certain limitations. Compared with BMI and WC, BRI seems to reflect abdominal obesity more accurately and effectively predict obesity-related metabolic chronic diseases (21). The study by Lucas et al. confirmed that metabolically unhealthy/obese individuals present a higher risk of developing CVD compared to metabolically healthy/obese individuals (22). BRI can significantly identify the presence of CMS (23). At the same time, BRI is a valid predictor of the risk of CVD (24). A cohort study in China found that individuals with intermediate and high BRI levels had a 22% and 55% higher risk of experiencing cardiovascular events, respectively, compared to those with low BRI (25). However, the relationship between BRI and the risk of CVD in patients with CMS in the United States (U.S.) remains underexplored. The above suggests that it would be positive to further explore the relationship between BRI and the occurrence of CVD among CMS patients.

Therefore, this study intends to investigate the association of BRI with the risk of CVD and its components including congestive heart failure (CHF), coronary heart disease (CHD), angina, heart attack, and stroke in patients with CMS in the U.S. In addition, we assessed the ability of the BRI to identify CVD and its components in the U.S. population. We hypothesized that the BRI would identify CVD better than BMI or WC.

## Materials and methods

### Study design

This cross-sectional investigation employed data from the NHANES implemented in the U.S. NHANES is a research initiative of the Centers for Disease Control and Prevention (CDC). It is designed to evaluate the health and nutritional status of the American population by means of comprehensive interviews and physical examinations. This survey protocol was approved by the Review Board of the National Center for Health Statistics (NCHS). All participants provided written informed consent. A detailed synopsis of the NHANES study and associated data can be found at https://www.cdc.gov/nchs/nhanes/.

### Study population

**Figure 1:**
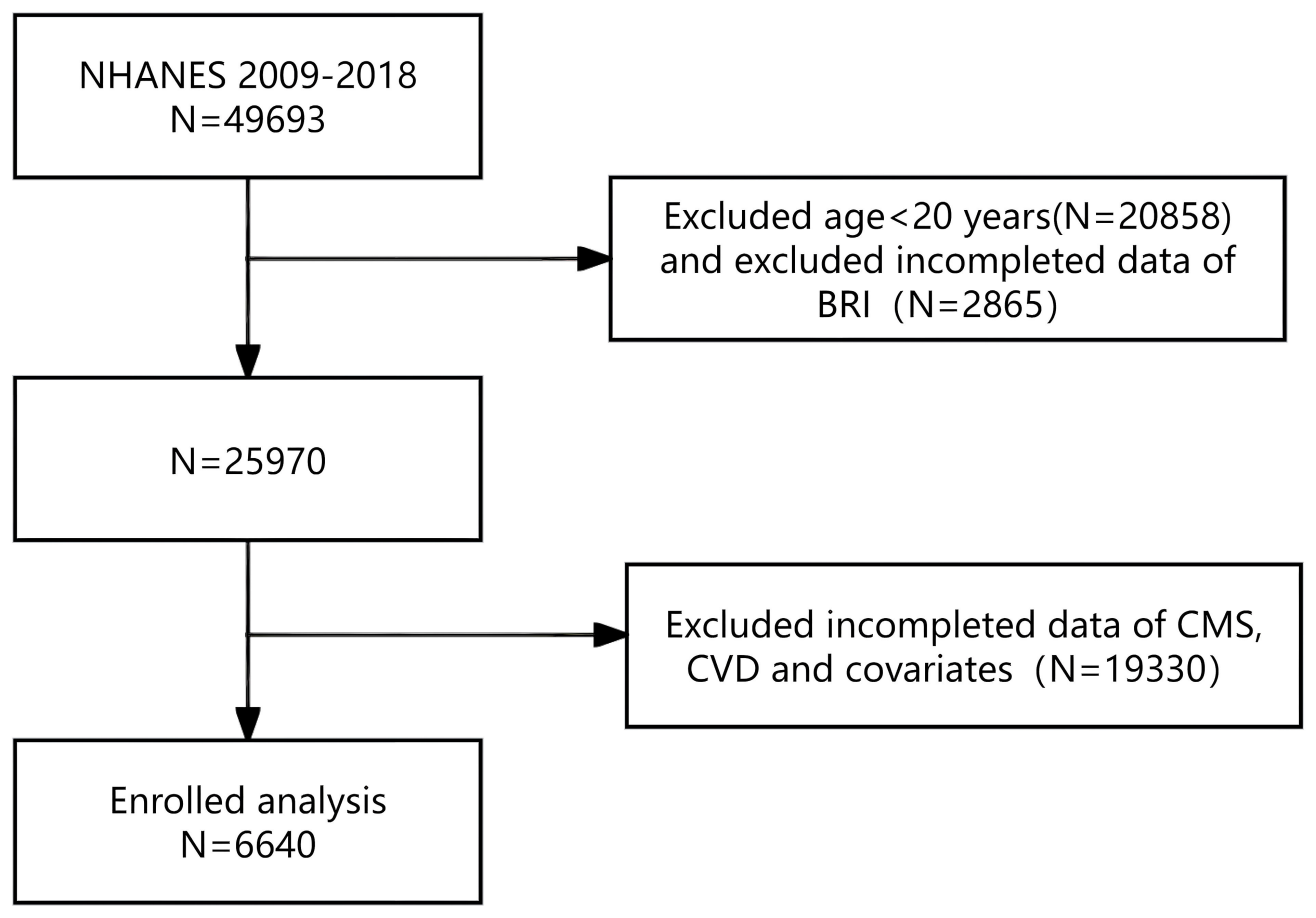
Screening flow of respondents.

The study encompassed subjects from the NHANES database covering the period from 2009 to 2018. These surveys offered extensive data on the BRI and multiple CVD, such as CHF, CHD, heart attack, angina, and stroke. Initially, 49,693 participants were included. After specific inclusion criteria were applied to filter the data, resulting in the exclusion of participants who met any of the following criteria: (1) age < 20 years; (2) missing data on height and WC data; (3) not diagnosed with CMS; (4) missing questionnaire data related to CVD; (5) missing data of covariates. Ultimately, a total of 6,640 subjects were included in the analysis (**Figure 1**).

### Measurement of the body roundness index

The BRI was utilized as an exposure factor and calculated using the following equation (18): BRI = 364.2 - 365.5 × (1- [WC (m)/2π]^2^/[0.5 × height (m)]^2^)^½^. Waist circumference and height can be found in the body measurements of the examination data.

### Measurement of cardiovascular disease

Data on cardiovascular conditions were obtained from the medical conditions section identified by the variable name prefix MCQ. This variable includes both self-reported and proxy-reported information that is collected through personal interviews regarding various health issues and histories for both children and adults. This section includes questions about whether a physician has diagnosed the participant with specific conditions, such as angina, congestive heart failure (CHF), coronary heart disease (CHD), heart attack, and stroke. Participants who responded ‘yes’ to these inquiries were categorized as having a history of CVD. We defined a composite endpoint for CVD. This endpoint included angina, congestive heart failure (CHF), coronary heart disease (CHD), heart attack, and stroke as primary outcomes. Meanwhile, the events related to these diseases were analyzed separately as secondary outcomes.

### Measurement of cardiometabolic syndrome

The diagnostic criteria for CMS were founded on the guidelines of the National Cholesterol Education Project (NCEP) Adult Treatment Panel III (ATP III) (9). CMS is characterized by the occurrence of three or more of the following conditions: (1) WC ≥ 102 cm for men and ≥ 88 cm for women; (2) Elevated serum TG ≥ 150 mg/dL; (3) HDL-C < 40 mg/dL for men and < 50 mg/dL for women; (4) Elevated fasting blood glucose ≥ 110 mg/dL; (5) Elevated blood pressure is defined as having a systolic blood pressure (SBP) of at least 130 mmHg or a diastolic blood pressure (DBP) of at least 85 mmHg, or for individuals who are currently taking oral antihypertensive medications. WC was collected using standard procedures during the physical examination. SBP and DBP were calculated as the arithmetic mean of up to four repeated measurements for each participant. TG and HDL-C were measured in serum, while fasting glucose was measured in plasma.

### Covariates

The covariates included in the analyses were age, sex, ethnicity, educational level, smoking status, diabetes, hypertension, BMI, SBP, and DBP. Laboratory test data comprised serum creatinine (SCR), serum uric acid (SUA), total cholesterol (TC), and HDL-C. Participants’ age were categorized as < 60 years and ≥ 60 years. Ethnicity classifications included Mexican American, Other Hispanic, Non-Hispanic White, Non-Hispanic Black, and Others. Educational attainment was categorized into three groups: below high school, high school or general educational development (GED), and above high school. Smoking status was classified as never smoked, previously smoked, and currently smoking; BMI was further categorized into normal, overweight, and obese groups. Hypertension was defined as a medical diagnosis of hypertension, intake of blood pressure-lowering drugs, or a sequence of three or more readings with systolic pressure at least 130 mmHg or diastolic pressure at least 80 mmHg (26). Diabetes mellitus defined as medically diagnosed or oral hypoglycemic drugs or insulin use. Each participant in the study received 3-4 blood pressure readings, and the blood pressure recordings represent the average of the readings.

### Statistical analysis

Participants were divided into quartiles (Q1 - Q4) according to their BRI values for analysis. Categorical variables were expressed as frequencies (percentages). Comparisons among groups were conducted using the chi-square test. Continuous variables were presented as mean ± standard deviation (SD). Evaluation was carried out using the t-test.

Multivariate logistic regression models were utilized to assess the connection between BRI and the primary outcome of CVD, as well as secondary outcomes including angina, CHF, CHD, heart attack, and stroke. Odds ratios (ORs) were calculated through three models. Model 1 had no adjustments made. Model 2 was adjusted for age, gender, and race. Model 3 included adjustments for age, gender, and race, education level, smoking status, diabetes, hypertension, BMI, SBP, and DBP. A generalized additive model (GAM) was employed for smooth curve fitting to examine potential non-linear associations between BRI and CVD. If the relationship was nonlinear, we estimated the threshold value and selected the infection point with the highest likelihood. Additionally, stratified logistic regression models were conducted to analyze subgroups based on age, sex, race, hypertension, diabetes, and BMI, with interaction tests employed to assess any variations in subgroup relationships. We reassessed the robustness of the results by multiple imputation for missing covariates mainly including HDL-C, TC, SCR, SUA, and BMI. Receiver operating characteristic (ROC) curves were used to assess the ability of BRI, WC and BMI in predicting CVD. Statistical analyses were carried out using R (version 4.4.1) and EmpowerStats (version 2.0). Statistical significance was defined as *P* < 0.05.

## Results

### Baseline characteristics of study participants

The study included 6,640 participants diagnosed with CMS, categorized according to BRI quartiles, as detailed in **Table 1**. The participants had a mean age of 56.72 ± 15.09 years, with females comprising 53.57% of the sample. The average BRI was 7.17± 2.27. The overall prevalence of CVD was 18.09%. This prevalence increased significantly as one moved across higher quartiles of BRI. Quartiles of higher BRI were linked with an elevated incidence of CHF, heart attack and stroke.

**Table 1:** Baseline characteristics of the study population.

Characteristics	BRI Index					
	Total	Q1	Q2	Q3	Q4	*P*-value
	n=6640	n=1660	n=1660	n=1659	n=1661	
Age (years)	56.72 ± 15.09	55.80 ± 15.21	58.01 ± 14.87	57.87 ± 15.25	55.21 ± 14.84	<0.001
Age group (years,%)						<0.001
<60	3414 (51.42)	913 (55.00)	804 (48.43)	779 (46.96)	918 (55.27)	
>=60	3226 (48.58)	747 (45.00)	856 (51.57)	880 (53.04)	743 (44.73)	
Gender (%)						<0.001
Male	3083 (46.43)	909 (54.76)	872 (52.53)	736 (44.36)	566 (34.08)	
Female	3557 (53.57)	751 (45.24)	788 (47.47)	923 (55.64)	1095 (65.92)	
Race (%)						<0.001
Mexican American	1104 (16.63)	201 (12.11)	324 (19.52)	294 (17.72)	285 (17.16)	
Other Hispanic	752 (11.33)	156 (9.40)	214 (12.89)	209 (12.60)	173 (10.42)	
Non-Hispanic White	2736 (41.20)	697 (41.99)	641 (38.61)	686 (41.35)	712 (42.87)	
Non-Hispanic Black	1397 (21.04)	322 (19.40)	316 (19.04)	351 (21.16)	408 (24.56)	
Other Race	651 (9.80)	284 (17.11)	165 (9.94)	119 (7.17)	83 (5.00)	
Education level (%)						<0.001
Less than high school	1881 (28.33)	412 (24.82)	501 (30.18)	526 (31.71)	442 (26.61)	
high school/GED	1589 (23.93)	395 (23.80)	394 (23.73)	380 (22.91)	420 (25.29)	
More than high school	3170 (47.74)	853 (51.39)	765 (46.08)	753 (45.39)	799 (48.10)	
BMI	33.27 ± 6.78	27.11 ± 2.94	30.45 ± 2.78	33.95 ± 3.30	41.58 ± 6.43	<0.001
BMI (%)						<0.001
Normal weight(<25)	417 (6.28)	384 (23.13)	32 (1.93%)	1 (0.06)	0 (0.00)	
Overweight (25~30)	1879 (28.30)	1001 (60.30)	706 (42.53)	165 (9.95)	7 (0.42)	
Obesity (>30)	4344 (65.42)	275 (16.57)	922 (55.54)	1493 (89.99)	1654 (99.58)	
SBP (mmHg)	131.90 ± 18.47	131.66 ± 17.98	132.42 ± 18.86	131.49 ± 18.38	132.02 ± 18.66	0.654
DBP (mmHg)	72.66 ± 12.80	73.50 ± 12.59	72.66 ± 12.43	72.51 ± 12.49	71.97 ± 13.62	0.002
SCR (mg/dL)	0.94 ± 0.49	0.94 ± 0.40	0.96 ± 0.65	0.94 ± 0.50	0.90 ± 0.37	0.005
SUA (umol/L)	351.03 ± 89.54	340.83 ± 88.59	345.55 ± 86.19	351.82 ± 87.30	365.92 ± 93.94	<0.001
TC (mg/dL)	192.10 ± 44.90	198.54 ± 49.00	192.35 ± 43.43	191.39 ± 44.15	186.13 ± 41.89	<0.001
HDL-C (mg/dL)	44.95 ± 13.24	45.28 ± 15.11	45.27 ± 12.92	44.84 ± 12.79	44.41 ± 11.94	0.486
Smoking status (%)						<0.001
Never	3436 (51.75)	850 (51.20)	861 (51.87)	861 (51.90)	864 (52.02)	
Former	1938 (29.19)	432 (26.02)	507 (30.54)	495 (29.84)	504 (30.34)	
now	1266 (19.07)	378 (22.77)	292 (17.59)	303 (18.26)	293 (17.64)	
Hypertension (%)						<0.001
Yes	4296 (64.70)	953 (57.41)	1078 (64.94)	1096 (66.06)	1169 (70.38)	
No	2344 (35.30)	707 (42.59)	582 (35.06)	563 (33.94)	492 (29.62)	
Diabetes (%)						<0.001
Yes	2169 (32.67)	390 (23.49)	519 (31.27)	592 (35.68)	668 (40.22)	
No	4471 (67.33)	1270 (76.51)	1141 (68.73)	1067 (64.32)	993 (59.78)	
CVD (%)						<0.001
Yes	1201 (18.09)	236 (14.22)	268 (16.14)	324 (19.53)	373 (22.46)	
No	5439 (81.91)	1424 (85.78)	1392 (83.86)	1335 (80.47)	1288 (77.54)	
CHF (%)						<0.001
Yes	403 (6.07)	69 (4.16)	78 (4.70)	106 (6.39)	150 (9.03)	
No	6237 (93.93)	1591 (95.84)	1582 (95.30)	1553 (93.61)	1511 (90.97)	
CHD (%)						0.001
Yes	475 (7.15)	85 (5.12)	117 (7.05)	137 (8.26)	136 (8.19)	
No	6165 (92.85)	1575 (94.88)	1543 (92.95)	1522 (91.74)	1525 (91.81)	
Angina (%)						<0.001
Yes	299 (4.50)	53 (3.19)	62 (3.73)	93 (5.61)	91 (5.48)	
No	6341 (95.50)	1607 (96.81)	1598 (96.27)	1566 (94.39)	1570 (94.52)	
Heart attack (%)						0.003
Yes	479 (7.21)	94 (5.66)	107 (6.45)	137 (8.26)	141 (8.49)	
No	6161 (92.79)	1566 (94.34)	1553 (93.55)	1522 (91.74)	1520 (91.51)	
Stroke (%)						0.072
Yes	394 (5.93)	77 (4.64)	102 (6.14)	105 (6.33)	110 (6.62)	
No	6246 (94.07)	1583 (95.36)	1558 (93.86)	1554 (93.67)	1551 (93.38)	

BRI, Body roundness index; GED, General educational Development; BMI, Body mass index; SBP, Systolic blood pressure; DBP, Diastolic blood pressure; SCR, Serum creatinine; SUA, Serum uric acid; TC, Total cholesterol; HDL-C, High-density lipoprotein cholesterol; CVD, Cardiovascular disease; CHF, Congestive heart failure; CHD, Congestive heart disease.

Obvious differences were detected among quartiles of BRI in terms of age, gender, race, education level, BMI, DBP, SCR, SUA, TC, smoking status, hypertension, diabetes, CHD, angina, and heart attack (*P* < 0.05). Participants in the highest quartile of BRI were more likely to have hypertension, diabetes, a higher obesity rate, and elevated SUA levels compared to those in the lowest quartile of BRI. No significant difference were found regarding SBP, HDL-C, or stroke among the quartiles.

### Association between BRI and the risk of CVD

#### Logistic regression analysis

Logistic regression analysis was carried out to assess the connection between BRI and both the primary outcome of CVD as well as secondary outcomes such as CHF, CHD, angina, heart attack, and stroke. The results from the three multivariate logistic regression models are presented in **Table 2**.

**Table 2:** The association between the BRI index and the risk of CVD.

Characteristic	Model 1		Model 2		Model 3	
	(OR95%CI)	*p*	(OR95%CI)	*p*	(OR95%CI)	*p*
CVD						
Continuous BRI	1.08 (1.05, 1.11)	**<0.0001**	1.14 (1.11, 1.18)	**<0.0001**	1.11 (1.07, 1.15)	**<0.0001**
BRI index quartile						
Q1	Reference		Reference		Reference	
Q2	1.16 (0.96, 1.40)	0.1219	1.08 (0.88, 1.32)	0.4682	1.02 (0.81, 1.29)	0.8781
Q3	1.46 (1.22, 1.76)	<0.0001	1.42 (1.17, 1.73)	0.0004	1.27 (0.97, 1.67)	0.0862
Q4	1.75 (1.46, 2.09)	<0.0001	2.13 (1.76, 2.59)	<0.0001	1.75 (1.31, 2.33)	**0.0001**
*P* for trend		<0.0001		<0.0001		**<0.0001**
CHF						
Continuous BRI	1.13 (1.09, 1.17)	**<0.0001**	1.19 (1.14, 1.25)	**<0.0001**	1.15 (1.08, 1.21)	**<0.0001**
BRI index quartile						
Q1	Reference		Reference		Reference	
Q2	1.14 (0.82, 1.58)	0.4479	1.04 (0.74, 1.46)	0.8110	0.93 (0.63, 1.38)	0.7311
Q3	1.57 (1.15, 2.15)	0.0043	1.48 (1.07, 2.03)	0.0166	1.15 (0.74, 1.79)	0.5293
Q4	2.29 (1.71, 3.07)	<0.0001	2.62 (1.93, 3.55)	<0.0001	1.82 (1.15, 2.87)	**0.0102**
P for trend		<0.0001		<0.0001		**<0.0001**
CHD						
Continuous BRI	1.05 (1.01, 1.09)	0.0113	1.13 (1.08, 1.18)	<0.0001	1.06 (1.00, 1.12)	0.0694
BRI index quartile						
Q1	Reference		Reference		Reference	
Q2	1.41 (1.05, 1.87)	0.0207	1.28 (0.95, 1.72)	0.1109	1.14 (0.81, 1.61)	0.4537
Q3	1.67 (1.26, 2.21)	0.0003	1.60 (1.19, 2.14)	0.0017	1.30 (0.87, 1.93)	0.1970
Q4	1.65 (1.25, 2.19)	0.0004	2.07 (1.54, 2.79)	<0.0001	1.46 (0.96, 2.23)	0.0797
P for trend		0.0008		<0.0001		0.0755
Angina						
Continuous BRI	1.08 (1.03, 1.13)	0.0011	1.12 (1.06, 1.18)	<0.0001	1.03 (0.97, 1.11)	0.3457
BRI index quartile						
Q1	Reference		Reference		Reference	
Q2	1.18 (0.81, 1.71)	0.3935	1.11 (0.76, 1.62)	0.5981	0.86 (0.56, 1.31)	0.4752
Q3	1.80 (1.28, 2.54)	0.0008	1.71 (1.21, 2.43)	0.0027	1.11 (0.69, 1.78)	0.6771
Q4	1.76 (1.24, 2.48)	0.0014	1.92 (1.35, 2.73)	0.0003	1.08 (0.65, 1.79)	0.7708
P for trend		0.0004		<0.0001		0.5331
Heart attack						
Continuous BRI	1.05 (1.01, 1.09)	0.0141	1.11 (1.07, 1.16)	<0.0001	1.05 (0.99, 1.11)	0.1101
BRI index quartile						
Q1	Reference		Reference		Reference	
Q2	1.15 (0.86, 1.53)	0.3444	1.05 (0.79, 1.42)	0.7215	0.96 (0.68, 1.35)	0.8052
Q3	1.50 (1.14, 1.97)	0.0035	1.48 (1.12, 1.96)	0.0065	1.16 (0.78, 1.71)	0.4678
Q4	1.55 (1.18, 2.03)	0.0016	1.92 (1.44, 2.54)	<0.0001	1.32 (0.88, 2.00)	0.1799
P for trend		0.0006		<0.0001		0.0657
Stroke						
Continuous BRI	1.03 (0.99, 1.08)	0.1574	1.05 (1.00, 1.10)	0.0526	1.02 (0.96, 1.09)	0.4412
BRI index quartile						
Q1	Reference		Reference		Reference	
Q2	1.35 (0.99, 1.82)	0.0555	1.29 (0.95, 1.76)	0.1037	1.48 (1.02, 2.15)	0.0379
Q3	1.39 (1.03, 1.88)	0.0331	1.30 (0.95, 1.77)	0.0966	1.44 (0.93, 2.23)	0.0987
Q4	1.46 (1.08, 1.97)	0.0136	1.49 (1.10, 2.03)	0.0111	1.54 (0.97, 2.44)	0.0647
P for trend		0.0278		0.0190		0.2428

Model 1: No adjustment.

Model 2: Adjusted for age, gender, race.

Model 3: Adjusted for age, gender, race, education level, BMI, SBP, DBP, smoking status, hypertension, diabetes.

OR, odds ratio; 95%CI, 95% Confidence interval.

Bold value indicates the statistical significance.

A consistent positive association was observed between elevated BRI levels and the heightened likelihood of CVD. In Model 1, without any adjustments, each 1-unit increase in BRI was associated with a 8% increase in the prevalence of CVD among participants with CMS (OR = 1.08, 95% CI 1.05 - 1.11, *P* < 0.0001). This relationship remained significant after adjustments for age, sex, and race in Model 2 (OR = 1.14, 95% CI 1.11 - 1.18, *P* < 0.0001), and further adjustments in Model 3, which accounted for age, gender, race, education level, BMI, SBP, DBP, smoking status, hypertension, diabetes (OR = 1.11, 95% CI 1.07 - 1.15, *P* < 0.0001). In the fully adjusted model 3, participants in the highest BRI quartile showed a 75% greater risk of CVD (OR = 1.75, 95% CI 1.31 - 2.33, *P* = 0.0001) compared to those in the lowest quartile of BRI.

No significant correlations were found for CHD, angina, heart attack, and stroke. (**Table 2**). Our investigation revealed a positive association between the BRI and an increased probability of the prevalence CHF among participants with CMS. In our unadjusted model 1, each 1-unit increase in BRI was associated with a 13% increase in the risk of CHF (OR = 1.13, 95% CI 1.09 - 1.17, *P* < 0.0001). This relationship remained significant after adjustments for age, sex, and race in Model 2 (OR = 1.19, 95%CI 1.14 - 1.25, *P* < 0.0001), and further adjustments in Model 3, which accounted for age, gender, race, education level, BMI, SBP, DBP, smoking status, hypertension, diabetes (OR = 1.15, 95% CI 1.08 - 1.21, *P* < 0.0001). After classifying the BRI into four quartiles, a significant statistical relationship was still observed. In comparison to individuals in the lowest quartile of BRI, those in the highest BRI quartile showed a 82% greater risk of CHF (Model 3: OR = 1.82, 95% CI 1.15 - 2.87, *P* = 0.0102).

Multiple imputation indicated that BRI remained positively associated with the risk of CVD and CHF in CMS patients, demonstrating robust results (**Supplementary Tables S1**).

#### Smooth curve analysis

**Figure 2:**
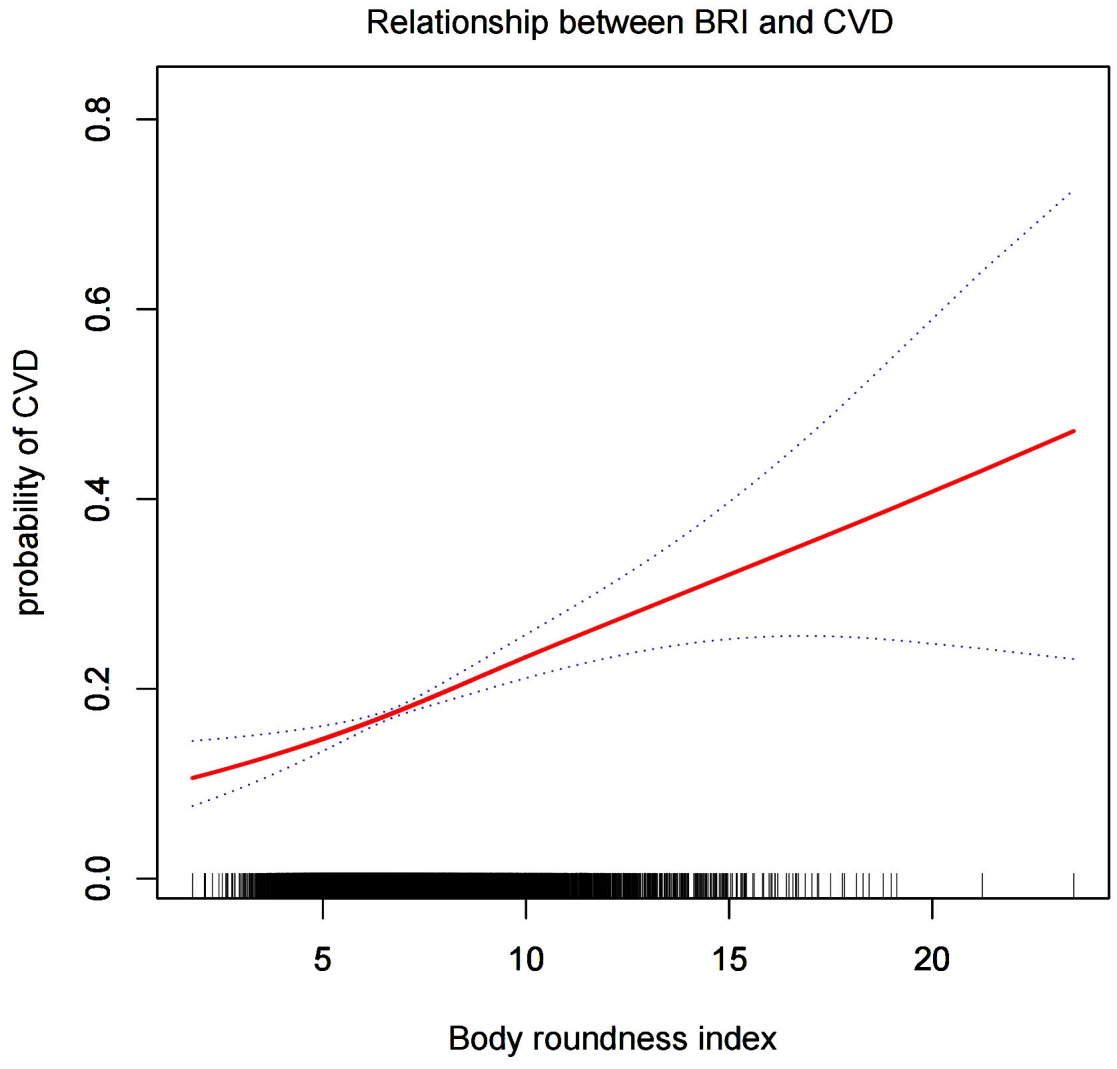
The smooth curve analysis between the BRI and the risk of CVD.

**Figure 3:**
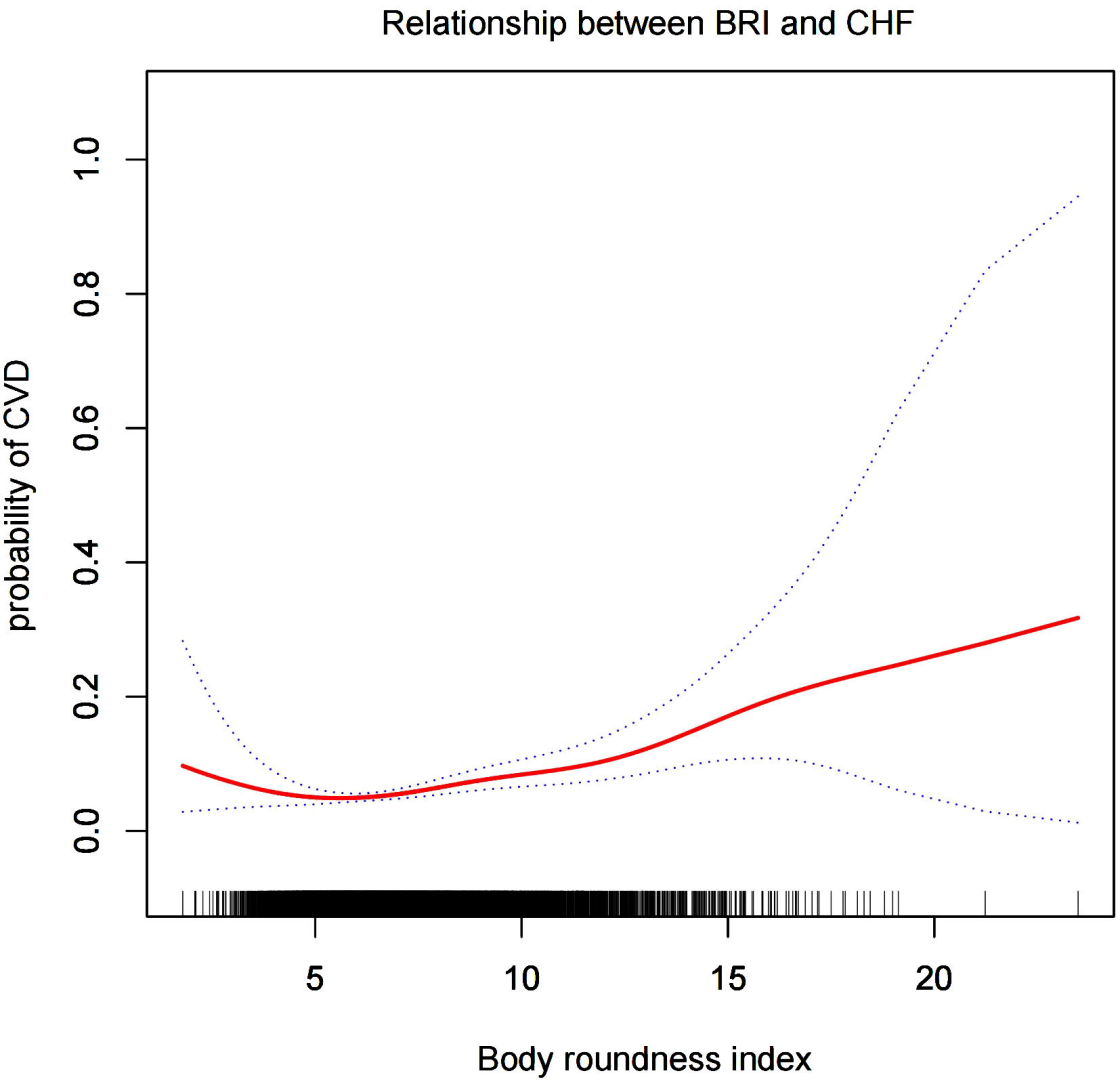
The smooth curve analysis between the BRI and the risk of CHF.

We conducted a smoothed curve-fitting analysis using GAM, which indicated that the BRI was linearly associated with the risk of CVD in CMS patients (**Figure 2**). However, the association between BRI and CHF were revealed to be a U-shaped association (**Figure 3**). And then, the threshold effects analysis showed a BRI cut-off of 4.53 units, and showed that when the BRI level was lower than 4.53, the prevalence of CHF decreased by 58% for every 1-unit decrease in BRI (OR = 0.42, 95% CI 0.22 - 0.79, *P* = 0.0072), but after this inflection point, the prevalence of CHF increased by 16% for every 1-unit increase in BRI (OR = 1.16, 95% CI 1.09 - 1.22, *P <*0.0001) (**Table 3**).

**Table 3:** Analysis of the threshold effect between BRI and the risk of CHF.

Threshold effect analysis	CHF
	(OR95%CI) *P*-value
BRI	
Inflection point of BRI (K)	4.53
<K slope	0.42 (0.22, 0.79) 0.0072
>K slope	1.16 (1.09, 1.22) <0.0001
Log-likelihood ratio test	0.004

#### Subgroup analysis

Stratification and interaction analysis were conducted for age, gender, race, hypertension, diabetes and BMI to further investigate the association between BRI and the risk of CVD and CHF among CMS patients. **Table 4** and **Table 5** demonstrated that the relationship between BRI and CVD was consistent across the various subgroups. There was no significant interaction effect between BRI and stratified variables (*P* for interaction > 0.05). Notably, BMI interacted with the association (*P* for interaction = 0.0118), with BRI being associated with decreased risk of CHF in a subgroup of normal weight subjects (OR = 0.45, 95% CI 0.24 - 0.86, *P* = 0.0154) and increased risk of CHF in a subgroup of obese subjects (OR = 1.16, 95% CI 1.10 - 1.23, *P* = 0.0154). Age, gender, race, hypertension, and diabetes did not influence the association (*P* for interaction > 0.05).

**Table 4:** Subgroup analysis for the association between BRI and CVD.

Subgroup	OR (95%CI)	*P*-value	*P* for interaction
Age			0.2933
<60	1.07 (1.02, 1.13)	0.0103	
≥60	1.12 (1.06, 1.18)	<0.0001	
Gender			0.4572
Male	1.13 (1.07, 1.19)	<0.0001	
Female	1.10 (1.05, 1.15)	<0.0001	
Race			0.6111
Mexican American	1.09 (0.97, 1.22)	0.1471	
Other Hispanic	1.19 (1.04, 1.35)	0.0114	
Non-Hispanic White	1.12 (1.06, 1.19)	0.0002	
Non-Hispanic Black	1.07 (1.00, 1.15)	0.0666	
Other Race	1.18 (0.99, 1.40)	0.0601	
Hypertension			0.8028
Yes	1.11 (1.06, 1.15)	<0.0001	
No	1.12 (1.02, 1.23)	0.0196	
Diabetes			0.7735
Yes	1.10 (1.05, 1.16)	0.0004	
No	1.12 (1.06, 1.18)	<0.0001	
BMI			0.6305
Normal weight	0.98 (0.67, 1.45)	0.9350	
Overweight	1.18 (1.00, 1.40)	0.0495	
Obesity	1.11 (1.07, 1.16)	<0.0001	

This analysis was adjusted for age, gender, race, education level, BMI, SBP, DBP, smoking status, hypertension, and diabetes.

**Table 5:** Subgroup analysis for the association between BRI and CHF.

Subgroup	OR (95%CI)	*P*-value	*P* for interaction
Age			0.6294
<60	1.12 (1.03, 1.21)	0.0098	
≥60	1.15 (1.07, 1.23)	0.0002	
Gender			0.5115
Male	1.17 (1.08, 1.26)	<0.0001	
Female	1.13 (1.06, 1.21)	0.0003	
Race			0.4228
Mexican American	1.09 (0.90, 1.32)	0.3599	
Other Hispanic	1.06 (0.88, 1.29)	0.5468	
Non-Hispanic White	1.17 (1.08, 1.26)	0.0002	
Non-Hispanic Black	1.12 (1.01, 1.24)	0.0303	
Other Race	1.40 (1.10, 1.78)	0.0060	
Hypertension			0.5639
Yes	1.15 (1.09, 1.22)	<0.0001	
No	1.08 (0.89, 1.32)	0.4400	
Diabetes			0.8127
Yes	1.14 (1.06, 1.22)	0.0004	
No	1.15 (1.06, 1.25)	0.0008	
BMI			0.0118
Normal weight	0.45 (0.24, 0.86)	0.0154	
Overweight	1.12 (0.85, 1.47)	0.4189	
Obesity	1.16 (1.10, 1.23)	<0.0001	

This analysis was adjusted for age, gender, race, education level, BMI, SBP, DBP, smoking status, hypertension, and diabetes.

### BRI as a predictor for CVD and CHF

**Figure 4:**
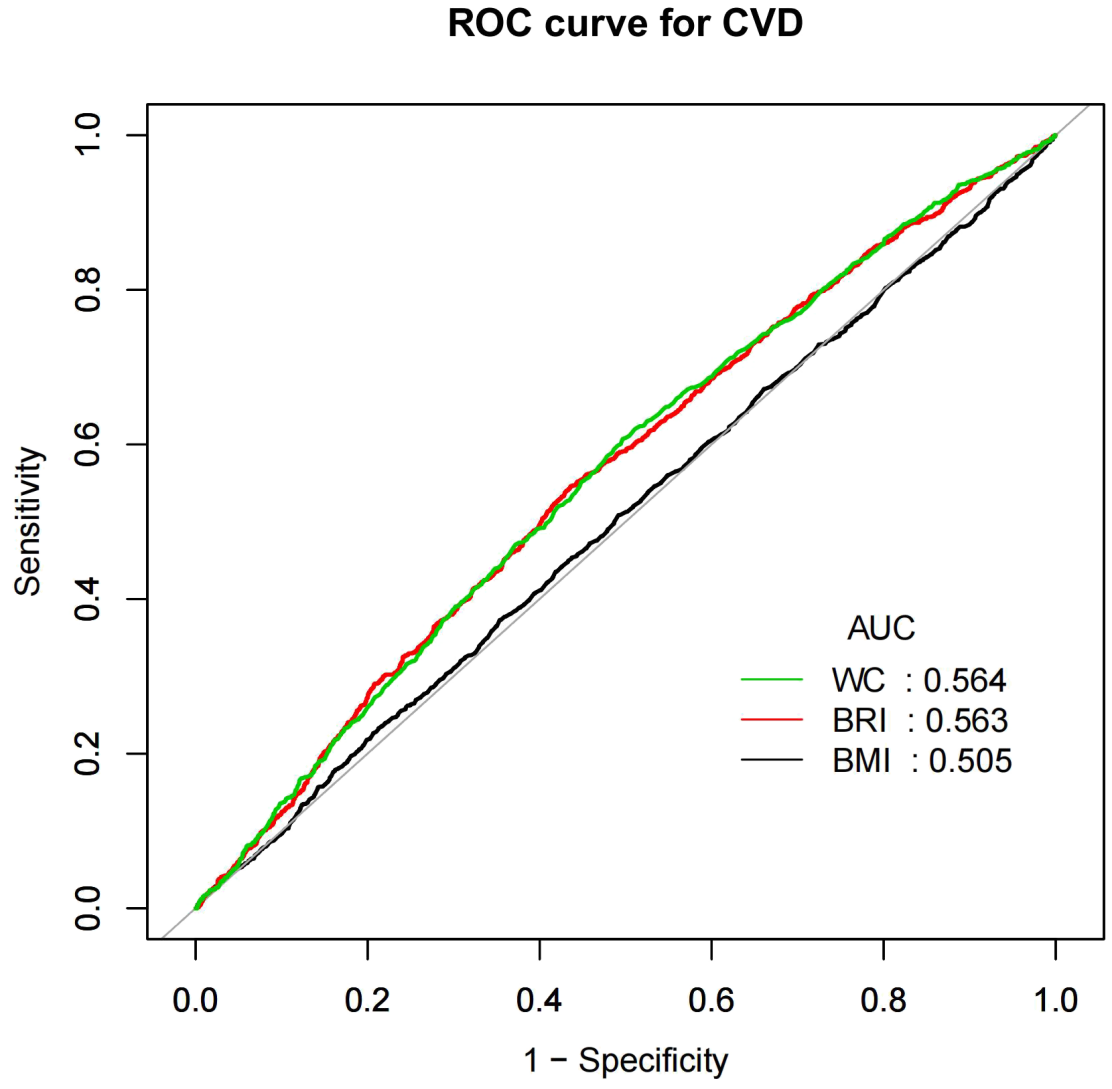
CVD, Cardiovascular disease; WC, Waist; BRI, Body roundness index; BMI, Body mass index; AUC, Area under the curve; ROC, Receiver operating characteristic.

**Figure 5:**
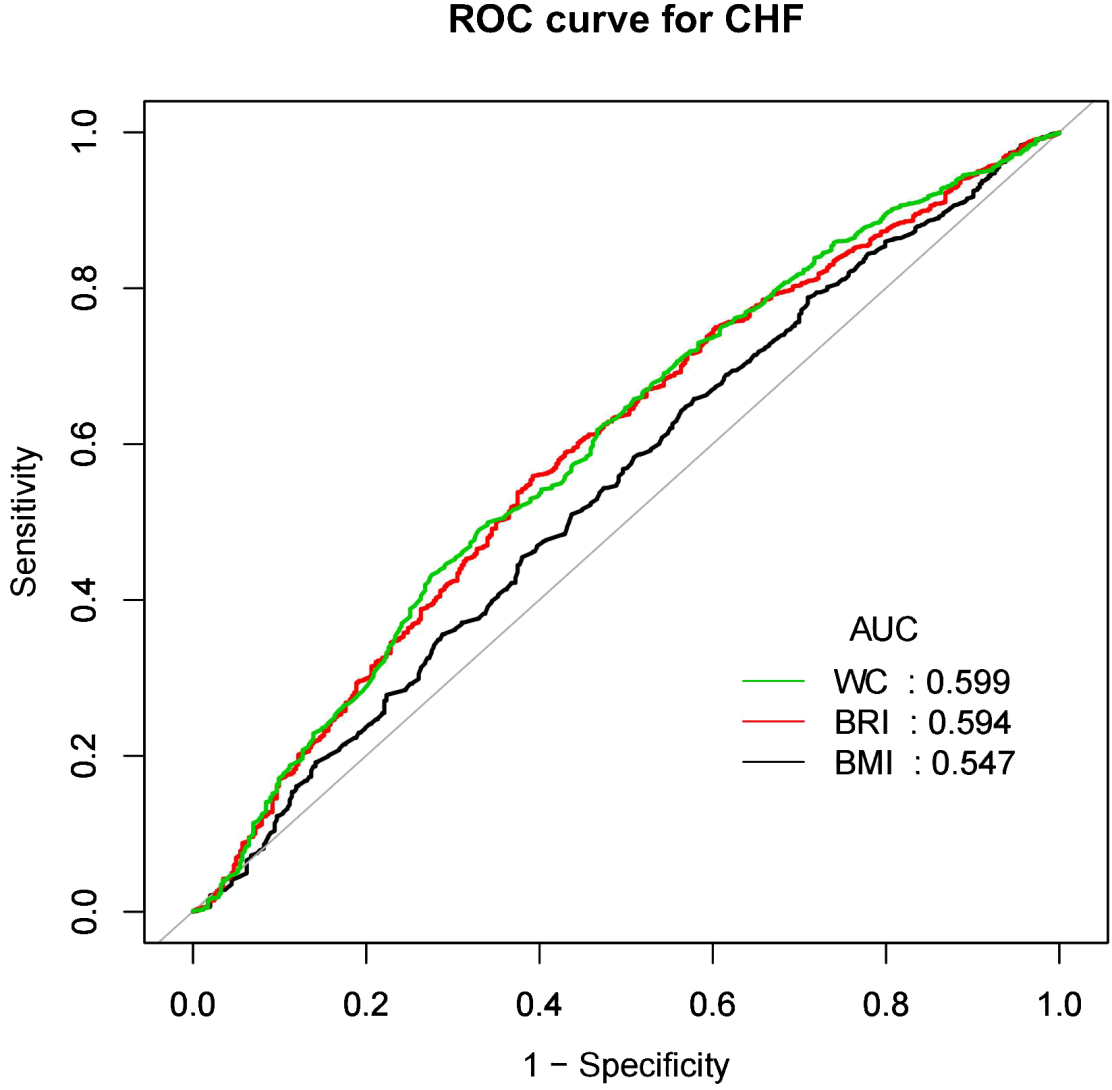
CHF, Congestive heart failure; WC, Waist; BRI, Body roundness index; BMI, Body mass index; AUC, Area under the curve; ROC, Receiver operating characteristic.

**Figures 4** and **5** showed the area under the curve (AUC) of diagnostic capability in the CVD and CHF. These results revealed that both BRI, WC and BRI had statistically significant diagnostic capability for the detection of CVD and CHF (AUC > 0.5). BRI has diagnostic capability in detecting CVD and CHF Superior to BMI (for CVD: AUC = 0.563, for CHF: AUC = 0.594), but not better than WC (for CVD: AUC = 0.564, for CHF: AUC = 0.599).

## Discussion

This study, which encompassed 6,640 participants, detected a positive correlation between a high BRI and an elevated risk of CVD among adults with CMS in the U.S.

Additionally, our results reveal a nonlinear relationship between BRI and CVD risk, whereas a u-shaped correlation was observed with the risk of CHF. When the BRI level was lower than 4.53, the risk of CHF decreased by 58% for every 1-unit decrease in BRI, but after this inflection point, the risk increased by 16% for every 1-unit increase in BRI. The association between BRI and CVD was consistent across subgroups and there was no significant interaction. However, BMI interacted with the association, with BRI being associated with decreased risk of CHF in a subgroup of normal weight subjects and increased risk of CHF in a subgroup of obese subjects. Multiple imputation suggests proof of the robustness of the findings. More importantly, BRI has similar ability to predict CVD and CHF as WC, and better than BMI.

Previous studies have focused on the potential role of BRI in cardiovascular disease morbidity and mortality or cardiometabolic risk factors (23, 25, 27). BRI has been shown to be a valid predictor of cardiometabolic risk and is significantly associated with cumulative cardiometabolic risk factors (23, 27, 28). Cohort studies in China have shown that a long-term increase in BRI is associated with an increased risk of CVD, stroke, or cardiac events (25, 29). Moreover, Li et al. demonstrated that the BRI was superior to other anthropometric measures, including waist circumference, in predicting the risk of cardiovascular disease in a Chinese population (24). However, Maessen et al. showed that the BRI was not superior as a novel body metric for identifying CVD compared to established anthropometric measures such as BMI and WC (30). Higher WC is associated with an increased risk of CVD, and the positive association between WC and new myocardial infarction and stroke has been shown to be higher in individuals <60 years of age (31). Our conclusions are consistent with previous studies that a higher BRI is associated with an increased risk of CVD. Also, BRI predicted CVD similarly to WC but better than BMI. Unusually, we also found a U-shaped association between BRI and CHF in participants evaluated for CMS, which has not been found in previous studies. This may be since previous studies focused on cardiovascular diseases such as stroke and myocardial infarction. However, the low prevalence of CHF in this study encourages future studies to further explore the potential nonlinear association between BRI and CHF. In addition, our study found that BRI was associated with a reduced risk of CHF in a subgroup of normal-weight subjects and an increased risk of CHF in a subgroup of obese subjects. There is a complex link between BMI and CHF that may involve multiple aspects. Higher BMI may increase the risk of CHF through melatonin secretion and metabolism, reduced sleep quality, and reduced activity (32). In the Belgian cohort, patients in the high inositol level group (highest tertile) also had a higher BMI, predicting poor clinical outcomes in heart failure patients with preserved ejection fraction (HFpEF) (33). This may be related to IR or other metabolic disorders due to obesity, which in turn affects inositol metabolism. This suggests that weight control may help reduce the risk of heart failure and improve prognosis. Another study suggested that machine learning algorithms may help to address the heterogeneity of HFpEF patients and provide new directions for precision therapy (34). This deserves further exploration.

Traditional indicators like BMI and WC have limitations in clinical scenarios. BMI does not distinguish between fat, muscle, and bone mass. Meanwhile, WC measures abdominal fat without taking height into consideration, resulting in potential misclassification of obesity in individuals with different heights (35). In contrast, the BRI uses an ellipsoid model to assess body size by integrating waist circumference and height independent of body weight, allowing for a more accurate representation of body fat as a proportion of total body weight (18). And it is critical for identifying health risks in individuals with an abnormal distribution of body fat (36). BRI effectively quantifies central obesity and is significantly associated with metabolic dysregulation, inflammatory response, vascular dysfunction, and oxidative stress, all of which can accelerate the progression of CVD (37). A study showed that the BRI performed similarly or better than BMI and WC in predicting CMS and CMS components in Peruvian adults, and showed the best ability to identify IR in obese and overweight populations (38). The mechanisms linking elevated BRI levels to increased CVD risk are not fully understood in patients with CMS. We will elaborate on the following aspects. First, previous cross-sectional studies have demonstrated a role for branched-chain amino acids (BCAAs) and lipid metabolism in the pathogenesis of type 2 diabetes mellitus (T2D) and coronary atherosclerotic heart disease (CAD), suggesting that obesity has an important role in the development of CVD (39). Secondly, the accumulation of excess body fat contributes to an inflammatory response in the blood and leads to oxidative stress, which increases the risk of CVD (17). This suggests that by calculating the BRI, we can identify the risk of metabolism in overweight and obese people at an early stage and reduce the risk of CVD by controlling weight and reducing fat accumulation. In recent years, an increasing number of studies have focused on the association between gut fungi and metabolism (40). This suggests that the relationship between gut fungi and other metabolic diseases, such as diabetes and cardiovascular disease, deserves further investigation. Kun et al. showed that gut fungi may affect CAD through mechanisms such as influencing immunity, metabolic processes, or systemic inflammation (41). Therefore, it is promising to develop interventional strategies based on gut fungi in the future, e.g., by modulating the intestinal fungal community for the prevention and treatment of metabolic diseases.

## Limitation

There are several limitations of our study. Firstly, being a cross-sectional study, it cannot establish a causal connection between BRI and the risk of CVD in patients with CMS. This design is unable to track changes in variables over time or recognize trends and dynamic patterns in time-series data, thus limiting its ability to assess long-term impacts and changes in time dynamics. In future studies, prospective cohort studies could be used to validate our findings. Secondly, diagnosis of CVD relied on participant self-reporting, but participants’ lack of knowledge about cardiovascular disease could affect the accuracy of subsequent analyses. Thus, the BRI’s ability to recognize CVD may be somewhat higher. In addition, the research sample is largely composed of subjects originating from the US., which limits the applicability of the findings to a worldwide context. Although we have strived to control for potential complicating variables, the likelihood of remaining confounding factors cannot be entirely ruled out (such as dietary habits and physical activity levels). Undocumented eating habits may affect body weight and fat distribution, which in turn affects BRI. Physical activity not only affects body weight and shape, but also directly affects cardiovascular health. Physical inactivity may lead to a higher risk of BRI and CVD, but this confounder may not be adequately captured in the NHANES data. Future studies should consider a more comprehensive assessment and adjustment of these potential confounders. Furthermore, we did not account for the influence of pharmacological treatments. Patients with CMS often experience multiple coexisting chronic diseases that necessitate antihypertensive, hypoglycemic, and lipid-lowering therapies. This could lead to a decrease in the number of patients we have diagnosed with CMS. Despite these limitations, this study highlights the association between BRI and the risk of cardiovascular disease in patients with CMS and paves the way for future longitudinal studies.

## Conclusion

This study demonstrated the positive relationship between the BRI and CVD in individuals diagnosed with CMS. Notably, a U-shaped correlation was observed between BRI and the risk of CHF. Increased assessment of BRI would facilitate easier and more effective screening of individuals at risk for CHF. In addition, this threshold could be used as a target for interventions to reduce CHF risk. Future studies should further explore whether interventions targeting the BRI may improve the clinical prognosis of CMS patients with CVD.
