# Supplementary material for: Association of body roundness index with cardiovascular disease in patients with cardiometabolic syndrome: a cross-sectional study based on NHANES 2009-2018
Source: Front Endocrinol (Lausanne). 2025 Feb 3;16:1524352. doi: 10.3389/fendo.2025.1524352 (PMC11830584; doi:10.3389/fendo.2025.1524352)
Supplement: Supplementary file 1 [file DataSheet1.pdf]

Supplementary Material

Table S1 The association between the BRI index and the risk of CVD.

Table S1 The association between the BRI index and the risk of CVD.

Characteristic	Model 1		Model 2		Model 3	
	(OR95%CI)	*p*	(OR95%CI)	*p*	(OR95%CI)	*p*
			**CVD**			
Continuous BRI	1.08 (1.05, 1.11)	<0.0001	1.14(1.11, 1.17)	<0.0001	1.10 (1.06, 1.14)	**<0.0001**
BRI index quartile						
Q1	Reference		Reference		Reference	
Q2	1.18 (0.98, 1.42)	0.0851	1.10 (0.90, 1.34)	0.3593	1.03 (0.81, 1.29)	0.8240
Q3	1.50 (1.25, 1.80)	<0.0001	1.45 (1.20, 1.76)	0.0002	1.26 (0.97, 1.65)	0.0875
Q4	1.75 (1.46, 2.09)	<0.0001	2.15 (1.77, 2.60)	<0.0001	1.71 (1.29, 2.27)	**0.0002**
P for trend		<0.0001		<0.0001		**<0.0001**
			**CHF**			
Continuous BRI	1.12 (1.08, 1.17)	<0.0001	1.18 (1.13, 1.23)	<0.0001	1.13 (1.07, 1.19)	**<0.0001**
BRI index quartile						
Q1	Reference		Reference		Reference	
Q2	1.15 (0.83, 1.59)	0.4077	1.05 (0.76, 1.47)	0.7576	0.92 (0.63, 1.35)	0.6724
Q3	1.63 (1.20, 2.20)	0.0017	1.51 (1.11, 2.06)	0.0090	1.15 (0.75, 1.76)	0.5359
Q4	2.19 (1.64, 2.92)	<0.0001	2.49 (1.85, 3.37)	<0.0001	1.68 (1.07, 2.61)	**0.0227**
P for trend		<0.0001		<0.0001		**0.0005**
			**CHD**			
Continuous BRI	1.05 (1.01, 1.09)	0.0143	1.12 (1.08, 1.17)	<0.0001	1.05 (0.99, 1.12)	0.0922
BRI index quartile						
Q1	Reference		Reference		Reference	
Q2	1.41 (1.06, 1.88)	0.0177	1.29 (0.96, 1.74)	0.0906	1.18 (0.84, 1.66)	0.3451
Q3	1.75 (1.33, 2.31)	<0.0001	1.68 (1.26, 2.24)	0.0004	1.40 (0.94, 2.07)	0.0971
Q4	1.65 (1.25, 2.17)	0.0005	2.08 (1.55, 2.79)	<0.0001	1.50 (0.98, 2.28)	0.0592
P for trend		0.0008		<0.0001		0.0781

			**Angina**			
Continuous BRI	1.08 (1.03, 1.13)	0.0018	1.12 (1.06, 1.17)	<0.0001	1.03 (0.96, 1.10)	0.4662
BRI index quartile						
Q1	Reference		Reference		Reference	
Q2	1.19 (0.83, 1.72)	0.3477	1.12 (0.77, 1.63)	0.5482	0.87 (0.57, 1.33)	0.5210
Q3	1.87 (1.33, 2.62)	0.0003	1.76 (1.24, 2.48)	0.0014	1.13 (0.71, 1.81)	0.6020
Q4	1.74 (1.24, 2.46)	0.0015	1.91 (1.35, 2.72)	0.0003	1.07 (0.65, 1.76)	0.7895
P for trend		0.0004		<0.0001		0.6103
			**Heart attack**			
Continuous BRI	1.05 (1.01, 1.09)	0.0110	1.11 (1.07, 1.16)	<0.0001	1.04 (0.98, 1.10)	0.1569
BRI index quartile						
Q1	Reference		Reference		Reference	
Q2	1.21 (0.91, 1.60)	0.1947	1.11 (0.83, 1.49)	0.4727	1.01 (0.72, 1.41)	0.9672
Q3	1.62 (1.24, 2.12)	0.0004	1.60 (1.21, 2.11)	0.0010	1.23 (0.84, 1.81)	0.2864
Q4	1.58 (1.21, 2.07)	0.0008	1.97 (1.49, 2.61)	<0.0001	1.33 (0.89, 2.00)	0.1648
P for trend		0.0004		<0.0001		0.0954
			**Stroke**			
Continuous BRI	1.04 (0.99, 1.08)	0.0851	1.06 (1.01, 1.11)	0.0152	1.03 (0.97, 1.10)	0.3052
BRI index quartile						
Q1	Reference		Reference		Reference	
Q2	1.33 (0.99, 1.80)	0.0589	1.28 (0.95, 1.74)	0.1084	1.44 (1.00, 2.08)	0.0497
Q3	1.36 (1.01, 1.83)	0.0425	1.27 (0.93, 1.72)	0.1270	1.34 (0.87, 2.06)	0.1806
Q4	1.51 (1.13, 2.02)	0.0057	1.58 (1.17, 2.14)	0.0029	1.54 (0.98, 2.41)	0.0591
P for trend		0.0113		0.0046		0.1664

Model 1: No adjustment. Model 2: Adjusted for age, gender, race

Model 3: Adjusted for age, gender, race, education level, BMI, SBP, DBP, smoking status, hypertension, diabetes

OR, odds ratio; 95%CI, 95% Confidence interval

Bold value indicates the statistical significance
